# Evaluation of the Effectiveness of Advanced Technology Clinical Simulation Manikins in Improving the Capability of Australian Paramedics to Deliver High-Quality Cardiopulmonary Resuscitation: Pre- and Postintervention Study

**DOI:** 10.2196/49895

**Published:** 2024-12-24

**Authors:** Alison Zucca, Jamie Bryant, Jeffrey Purse, Stuart Szwec, Robert Sanson-Fisher, Lucy Leigh, Mike Richer, Alan Morrison

**Affiliations:** 1 School of Medicine and Public Health, University of Newcastle, Newcastle, Australia; 2 Equity in Health and Wellbeing Research Program, Hunter Medical Research Institute, New Lambton Heights, Australia; 3 New South Wales Ambulance, New South Wales Health, Sydney Olympic Park, Australia; 4 Data Sciences, Hunter Medical Research Institute, Newcastle, Australia

**Keywords:** paramedicine, cardiopulmonary resuscitation, clinical simulation, professional development, manikins, effectiveness, technology, paramedics, patient care, simulation-based training, deployment

## Abstract

**Background:**

Emergency medical services attend out-of-hospital cardiac arrests all across Australia. Resuscitation by emergency medical services is attempted in nearly half of all cases. However, resuscitation skills can degrade over time without adequate exposure, which negatively impacts patient survival. Consequently, for paramedics working in areas with low out-of-hospital cardiac arrest case volumes, ambulance services and professional bodies recognize the importance of alternative ways to maintain resuscitation skills. Simulation-based training via resuscitation manikins offers a potential solution for maintaining paramedic clinical practice skills.

**Objective:**

The aim of the study is to examine the effectiveness of advanced technology clinical simulation manikins and accompanying simulation resources (targeted clinical scenarios and debriefing tools) in improving the demonstrable capability of paramedics to deliver high-quality patient care, as measured by external cardiac compression (ECC) performance.

**Methods:**

A pre- and postintervention study design without a control group was used. Data were collected at the start of the manikin training forum (baseline), immediately following the training forum (time 2), and 6 to 11 months after the training forum (time 3). The study was conducted with paramedics from 95 NSW Ambulance locations (75 regional locations and 20 metropolitan locations). Eligible participants were paramedics who were employed by NSW Ambulance (N=106; 100% consent rate). As part of the intervention, paramedics attended a training session on the use of advanced technology simulation manikins. Manikins were then deployed to locations for further use. The main outcome measure was an overall compression score that was automatically recorded and calculated by the simulator manikin in 2-minute cycles. This score was derived from compressions that were fully released and with the correct hand position, adequate depth, and adequate rate.

**Results:**

A total of 106 (100% consent rate) paramedics participated, primarily representing regional ambulance locations (n= 75, 78.9%). ECC compression scores were on average 95% or above at all time points, suggesting high performance. No significant differences over time (*P*>.05) were identified for the overall ECC performance score, compressions fully released, compressions with adequate depth, or compressions with the correct hand position. However, paramedics had significantly lower odds (odds ratio 0.30, 95% CI 0.12-0.78) of achieving compressions with adequate rate at time 3 compared to time 2 (*P*=.01). Compressions were of a slower rate, with an average difference of 2.1 fewer compressions every minute.

**Conclusions:**

Despite this difference in compression rate over time, this did not cause significant detriment to overall ECC performance. Training and deployment of simulator manikins did not significantly change paramedics’ overall ECC performance. The high baseline performance (ceiling effect) of paramedics in this sample may have prevented the potential increase in skills and performance.

## Introduction

### Background

Across Australia, there were over 26,000 cases of out-of-hospital cardiac arrest (OHCA) attended by emergency medical services in 2019 [1], representing a crude incidence of 107.9 per 100,000 person-years. Resuscitation by emergency medical services is attempted in 44% of cases with an overall survival rate of approximately 13% [1].

Modifiable factors that can improve OHCA survival outcomes can include bystander-initiated cardiopulmonary resuscitation, faster emergency medical service response time, and quality of emergency medical services procedural care [2]. Paramedic OHCA case volume may be important to maintain quality resuscitation skills [3-7]. Dyson [3] found that the odds of survival were higher for patients treated by paramedics with 7 or more OHCA exposures compared to paramedics with 6 or fewer OHCA exposures during the preceding 3 years with a dose-response, whereby greater OHCA exposure was linked to greater odds of patient survival. Of note, there was no association between paramedic years of career experience and patient survival, highlighting the importance of case-volume exposures for all paramedics throughout their careers [8].

Conversely, resuscitation skills can degrade over time without adequate exposure to OHCA resuscitation [3] and negatively impact patient survival [3,9]. Paramedic exposure to OHCA is low, with the crude estimate of 2 or fewer resuscitations for OHCA per year in Victoria, Australia, and with significantly fewer OHCA exposures in paramedics placed in rural areas [10]. Anecdotal data from New South Wales and other Australian states suggest similarly low levels of resuscitations performed by paramedics working in rural and regional areas. Consequently, for paramedics practicing in areas with low OHCA case volumes, ambulance services and professional bodies recognize the importance of alternative ways to maintain resuscitation skills [11,12].

Simulation-based training via resuscitation manikins offers a potential solution for maintaining paramedic clinical practice skills in areas with low OHCA case volumes. Meta-analyses have demonstrated that simulation-based training for resuscitation using manikins is highly effective compared to no intervention and improves knowledge, skills, and patient outcomes across a range of health care professionals [11,12]. High-fidelity manikins that are technology-enhanced or computer-controlled have been found to be more beneficial than low-fidelity static manikins [8]. This may be because high-fidelity manikins more closely mimic clinical practice and allow the learner to physically interact with the simulated patient, assess physical findings, and make clinical decisions [13]. Furthermore, simulation-based training is more effective when it includes the additional elements of regular booster practice, team or group dynamics, distraction via noise or external stressors, and integrated feedback [11].

### Simulated Curriculum for Out-of-Hospital Paramedic Education Project

The availability of simulated education technology provides an opportunity to implement high-quality in-location learning to support clinical capability, overcome access barriers, and move toward addressing modifiable factors in care delivery. Traditionally, NSW Ambulance has delivered most of its education and training opportunities to paramedics from a limited number of central and regional locations, including simulation resuscitation training using high-fidelity manikins. However, in 2017, a philanthropic gift allowed the service to purchase new education equipment via open market tender including advanced technology simulation manikins and accompanying clinical simulation resources (targeted clinical scenarios and debrief tools) for each location. These resources were called the Simulated Curriculum for Out of Hospital Paramedic Education (SCOPE) resources. The purchase of the SCOPE resources in 2019 was supported by train-the-trainer style forums (SCOPE forums), whereby a paramedic at each site was instructed to use the manikin and teach their location colleagues; a New South Wales technical manager; a paramedic educator; and a project manager to assist with the rollout of the resources. Simulation manikins were deployed at every location across New South Wales to allow them to be accessed at times that suit the paramedic to practice a variety of resuscitation, trauma, cardiac, and medical scenarios, including presentations that may not be regularly experienced in the field.

The implementation of SCOPE across New South Wales represented an important opportunity to evaluate the impact of this unique technology in enhancing the capability of the New South Wales paramedic workforce in OHCA. In this context, the advanced technology simulation manikins are being used as a professional development activity for skills maintenance with registered paramedics rather than a skills acquisition activity with trainees. No Australian studies have been conducted with registered paramedics to evaluate the impact of simulation manikins on paramedic resuscitation skills. Previous Australian and international studies have been limited to samples of health care professionals practicing in tertiary hospital settings [11] or to student paramedics for skills acquisition [13]. Of those studies that have included subsamples of paramedics, the number of paramedics constituted a small proportion of the total sample, and the results did not examine intervention effects by professional subgroups [14,15]. Consequently, results from this study can help to determine the role of advanced technology simulation manikins in cardiopulmonary resuscitation training and may be useful to optimize instructional design.

### Aims

This study aimed to examine the effectiveness of advanced technology clinical simulation manikins and training resources in improving the demonstrable capability of paramedics to deliver high-quality external cardiac compression (ECC) performance. We hypothesized that ECC performance would improve after training and be maintained over time among paramedics with access to the simulation training resources at their location.

## Methods

### Design

A pre- and postintervention study design without a control group was used [16]. The advanced technology clinical simulation manikins were deployed to southern and metropolitan NSW Ambulance locations of Australia across a 6-month period from September 29, 2020, to March 11, 2021. Data were collected at 3 time points: prior to the commencement of the manikin training forum (baseline), immediately following the manikin training forum (time 2: September 29, 2020, to March 11, 2021), and approximately 6 to 11 months after the manikin training forum (time 3: August 2021 to February 2022).

### Ethical Considerations

This study was approved by the Hunter New England Health Human Research Ethics Committee (reference 2019/ETH13379). Only individuals who provided written informed consent were included in the research. Participants could stop participating at any stage without giving a reason and without penalty and had the option to withdraw their data. All information provided to the research team by the SCOPE project manager was deidentified. No monetary compensation was provided to paramedics for their participation.

### Setting

NSW Ambulance operates from 226 locations located across metropolitan, regional, and rural areas with over 4900 paramedics. While all 226 locations received the intervention, 95 participated in this evaluation study. Participating locations were from the Southern Sector (60 locations), Inner Hunter Zone (15 locations), metropolitan superstations (10), and metropolitan central coast (10 locations). A total of 101 regional locations (Northern Sector, Western Sector, and New England Zone) did not participate in the evaluation, as the intervention resources had already been distributed; thus, a baseline measure could not be obtained. The remaining 30 metropolitan locations had not yet received the intervention resources.

### Intervention Description

The SCOPE project specifically aims to improve out-of-hospital care outcomes for patients. The primary objective is to extend the availability of peer-led clinical simulation resources to facilitate enhanced capability of the paramedic workforce. Central to this initiative are the SCOPE manikins (advanced technology simulation manikins, Laerdal Resusci Anne QCPR) and accompanying clinical simulation resources (targeted clinical scenarios and debrief tools). The manikins (Resusci Anne Advanced SkillTrainer [17]) are designed to simulate a range of life-threatening conditions and allow paramedics to treat the simulated patient and practice complex life-saving procedures.

To introduce SCOPE manikins and clinical simulation resources and train paramedics, a representative from each location (referred to as “champions”) was selected to attend a SCOPE forum. The location of the forums was selected to be within 2 hours of respective stations. Venues included regional ambulance training units, helicopter bases, and rural fire service headquarters. The 6-hour forum focused exclusively on instructing champions about how to use the manikins, care for and maintain the technology, how to conduct peer-led simulations, and debrief paramedics afterward using a modified SHARP debriefing tool [18]—the SHARPR to include referral process (R) for paramedics to a number of areas such as clinical training officers, station managers, and protocols for review. Instruction was delivered through written, verbal, and practical training methods by experienced paramedic educators who followed the same format when delivering each training forum. Since the SCOPE champions were qualified paramedics with prior experience in performing single-person ECC on both manikins and patients, practice sessions on the manikins before data collection were deemed unnecessary. Each SCOPE forum concluded with the data collection for the time 2 research phase, during which the participating champions once again conducted a cycle of 2 minutes of ECC.

At the conclusion of each forum, champions were provided with 1 manikin along with its accompanying clinical simulation resources to take back to their station for use. After the forum, champions were instructed to return to their locations and support and encourage their colleagues to engage with the manikin and clinical simulation resources. The manikins deployed at each location are accessible to all staff members, allowing paramedics to practice scenarios that they may not regularly encounter in the field. Reflecting real-world practice, the implementation of the SCOPE technology within each location was not standardized; instead, each station had the autonomy to use their station manikin according to their specific needs and preferences. For technology troubleshooting, paramedics were given the contact details of a SCOPE technical manager who could assist them over the phone or via teleconference.

### Participant Eligibility

Eligible paramedics were employed by NSW Ambulance, working in ambulance locations that were allocated advanced technology clinical simulation resources (ie, manikins) during the recruitment period (September 29, 2020, to March 11, 2021), and attended a SCOPE forum as a champion during the study recruitment period. Paramedics who attended a SCOPE forum outside of the study recruitment period were not included. Eligible paramedics were identified by a representative of the NSW Ambulance Service using available staff records. Champions either self-selected to be a part of the SCOPE project or were nominated by station leaders.

### Recruitment

At least 3 weeks prior to attendance at the SCOPE forum, champions were emailed a letter from the Director of Education of NSW Ambulance that informed them of the opportunity to participate in a research evaluation at the SCOPE forum. Champions were provided with a copy of the participant information statement that highlighted the data collected during the paramedic capability appraisal activity would remain confidential and deidentified (ie, no individual paramedic or location identifiable) and was for research purposes only. Champions were asked to consider whether they were willing for their deidentified data to be provided to the research team. At the start of the SCOPE forum, champions were invited to complete the consent form and return it to the research team indicating if they were willing for their deidentified data, collected via the simulator manikins as part of the paramedic capability appraisal activity, to be provided to the research team.

### Data Collection

A representative paramedic from the research team supervised data collection at each of the 3 time points. This occurred face-to-face during the forum for time 1 (morning session during the forum) and time 2 (afternoon session of the forum) and via videoconference at time 3. Data collected for each champion as part of the capability appraisal activity were recorded by the research team from the simulator manikin. Data were linked via an ID number for the 3 time points.

### Measures

#### Consent and Response Bias

A representative of the NSW Ambulance Service recorded the age and sex of all champions who attended the workshop to allow the determination of potential consent bias and response rates.

#### External Cardiac Compression

Single-person ECC performance was measured via an advanced technology simulator manikin (Laerdal Resusci Anne QCPR). At each time point, research participants were verbally instructed to perform 2 minutes of ECC. There were no formal practice sessions prior to each round of data collection. Time 1 data collection corresponded with participants’ first encounter with the new technology. Time 1 and time 2 data collection involved the use of the instructors’ manikin that had been previously set up at the forum. At time 3, participants set up their station manikin and associated technology for data collection and received a videoconference call from a paramedic who observed their ECC performance. Appraisal of performance was overseen by a paramedic. ECC performance was automatically recorded by the simulator manikin via the SimPad PLUS using an installed program called Basic Life Support Instructor. ECC can be efficiently recorded in 2-minute cycles (Laerdal). The software was able to provide participants and observers (paramedic educators) with real-time feedback on the performance for each of the following outcomes:

The specific outcome measures of ECC performance are mapped to evidence-based guidelines [19,20]. They are:

- Overall compression score: a weighted overall global score was automatically calculated by the SimPad PLUS via quality cardiopulmonary resuscitation algorithm that measures how close users are to the threshold for each compression throughout one 2-minute cycle. Greater weighting is applied to skills most important to patient survival (hand position) compared to other skills (hand release and compression depth). More detailed information on software scoring is present on the manufacturer’s website [21]. The greater the score, the better the performance (range 0%‐100%).
- Individual aspects of compression scores measured were (1) proportion of compressions with the correct hand position (range 0%‐100%), (2) proportion of compressions with adequate depth (chest compression 5‐cm to 6-cm depression; range 0%‐100%), (3) proportion of compressions fully released (fully releasing the patient’s chest before commencing the next compression; range 0%‐100%), and (4) proportion of compressions with adequate rate (chest compression rate of 100-120 compressions per minute; range 0%‐100%).

### Statistical Analysis Plan

All statistical analyses were programmed using SAS (version 9.4; SAS Institute Inc). Statistical significance was set a priori at *P*<.05. All data were checked for completeness and discrepancies before analysis. Descriptive statistics were used to summarize the patient age and sex. Demographic information is presented as mean (SD) and median (IQR) along with minimum and maximum values (range) for continuous variables. Categorical variables are presented as counts and proportions. ECC performance is presented as mean (SD) and median (IQR), and the proportion of perfect scores (a score of 100). To explore the change over time in each of the outcomes, mixed-effects ordinal logistic regression models were used, with random intercepts to account for the nonindependence of measurements from the same individual, and a fixed effect for the study time period. Odds ratios (ORs) of achieving a higher score between time points are reported with 95% CIs and *P* values. ORs >1 indicate increased odds in achieving a higher score between time periods, and ORs <1 indicate decreased odds of achieving a higher score between 2 time points. Finally, the generalized mixed modeling approach we used uses maximum likelihood estimation to handle missing outcome data under a missing at random assumption. We compared the demographics of nonresponders and responders at time 3 to assess the validity of assuming that the data were missing at random. If there were differential response rates across the demographics, we would have needed to impute the missing taking into account the demographics as well to ensure that the data remained missing at random for the models.

## Results

### Sample Characteristics

Overall, all 106 paramedics who attended the forum during the recruitment period were approached, and all (100%) consented. At time 1 and time 2, 12 data points were missing (for the same paramedics) due to software issues or uploading SimPad data to the server (time 2). At time 3, 78 values were missing because 13 participating paramedics relocated to a location without access to a SCOPE manikin, 9 were on extended leave (eg, long service, with injury, and parental leave), and many were too busy from responding to COVID-19 or could not work due to COVID-19 exposure and illness. Due to the large proportion of missing responses at the third time point, a comparison of demographics between responders and nonresponders at time 3 was conducted. At time 3, 28 (26.4%) participants responded, and 78 (73.6%) did not respond. No significant differences in age and sex were found between responders and nonresponders at time 3 (*P*_age_=.80 and *P*_sex_=.84).

Participant demographics are provided in Table 1. Most participants were male (n=66, 62.3%), with a mean age of 40.48 (SD 10.53) years. Participating paramedics were champions for 95 different ambulance locations.

**Table 1.** Paramedic participant characteristics at baseline (N=106).

Response		Values
**Age (years)**		
	Mean (SD)	40.48 (10.53)
	Range	24-65
**Sex, n (%)**		
	Male	66 (62.3)
	Female	40 (37.7)
**Locations (n=95), n (%)**		
	Metro	20 (21.1)
	Regional	75 (78.9)

### ECC Performance Over Time

Table 2 summarizes all the ECC performance measures before the SCOPE forum (time 1), immediately after the SCOPE forum training (time 2), and 6 to 11 months after the SCOPE forum (time 3). ECC performance scores were highly skewed toward a maximal score of 100%. Overall compression score had a mean score of 95 (SD 13.23) or above across all 3 time points. The proportion of paramedics with a perfect overall compression score was 21.3% (n=20) at time 1, 28.7% (n=27) at time 2, and 7.1% (n=2) at time 3.

**Table 2.** Summary of external cardiac compression (ECC) performance measures over time and proportions of perfect ECC performance scores.

ECC performance and response		Time (N=106)		
		Time 1 (baseline)	Time 2 (training)	Time 3
Missing, n		12	12	78
**Overall compression score**				
	Perfect score, n (%)	20 (21.3)	27 (28.7)	2 (7.1)
	Mean (SD)	95.00 (13.23)	97.23 (6.79)	98.61 (1.03)
	Median (IQR)	99 (98-99)	99 (99-100)	99 (98.5-99)
**Compressions with the correct hand position**				
	Perfect score, n (%)	74 (78.7)	80 (85.1)	25 (89.3)
	Mean (SD)	96.15 (12.81)	97.79 (7.90)	99.36 (1.91)
	Median (IQR)	100 (100-100)	100 (100-100)	100 (100-100)
**Compressions with adequate depth**				
	Perfect score, n (%)	80 (85.1)	79 (84.0)	25 (89.3)
	Mean (SD)	96.77 (13.03)	97.65 (10.74)	99.57 (1.57)
	Median (IQR)	100 (100-100)	100 (100-100)	100 (100-100)
**Compressions fully released**				
	Perfect score, n (%)	58 (61.7)	65 (69.1)	14 (50.0)
	Mean (SD)	91.49 (20.71)	94.29 (14.04)	89.75 (21.47)
	Median (IQR)	100 (96-100)	100 (99-100)	99.5 (87.5-100)
**Compressions with adequate rate**				
	Perfect score, n (%)	48 (51.1)	56 (59.6)	11 (39.3)
	Mean (SD)	87.77 (23.15)	90.53 (20.99)	85.71 (26.20)
	Median (IQR)	100 (90-100)	100 (98-100)	98 (86.5-100)
**Mean rate of compressions**				
	Mean (SD)	109.41 (6.57)	108.29 (5.77)	107.43 (6.72)
	Median (IQR)	109 (104-113)	109 (104-112)	106 (102.5-112)
**Mean compression depth (mm)**				
	Mean (SD)	60.97 (4.21)	61.17 (4.10)	61.21 (2.73)
	Median (IQR)	62 (58-64)	63 (59-64)	62 (59-63.5)

Looking at the individual measures of performance that determined the overall compression score, compressions with the correct hand position and compressions with adequate depth were the skills with the highest mean scores across all 3 time points. A perfect score for compressions with the correct hand position was achieved by 78.1% (n=74) of paramedics at time 1, 85.1% (n=80) at time 2, and 89.3% (n=25) at time 3. Similarly, compressions with adequate depth were achieved by 85.1% (n=80) of paramedics at time 1, 84% (n=79) at time 2, and 89.3% (n=25) at time 3.

The lowest performance scores were for compressions with adequate rate and compressions fully released (mean scores of 85 or above for time 1-time 3). A perfect score for compressions fully released was achieved by 61.7% (n=58) of paramedics at time 1, 69.1% (n=65) at time 2, and 50% (n=14) at time 3. A perfect score for compressions with adequate rate was achieved by 51.1% (n=48) at time 1, 59.6% (n=56) at time 2, and 39.3% (n=11) at time 3.

### Pre- and Postcomparison of ECC Performance

**Figure 1.**
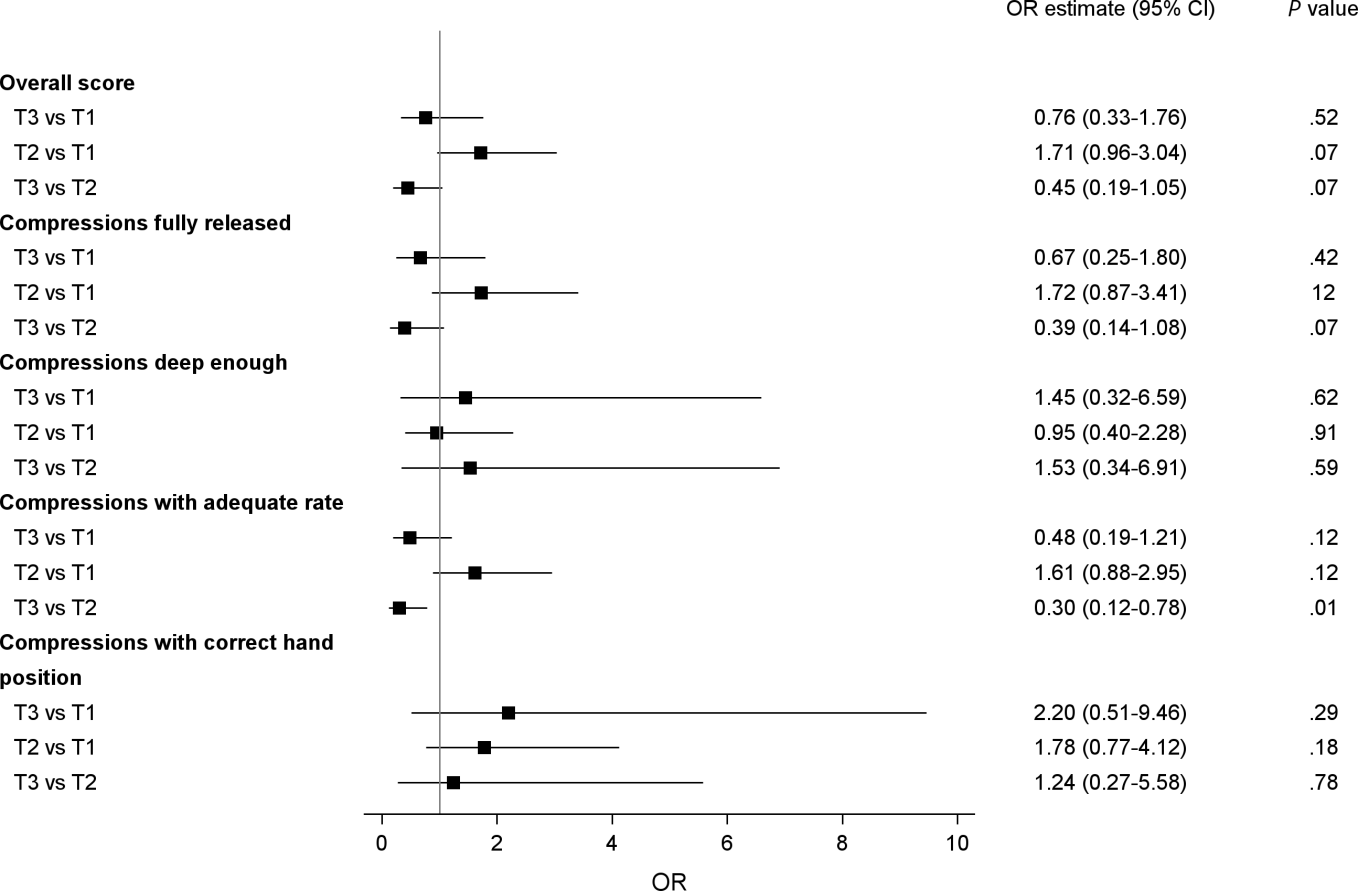
Mixed effects ordinal regression modeling for external cardiac compression performance measures. OR: odds ratio; T1: time 1; T2: time 2; T3: time 3.

Figure 1 presents comparisons over time using mixed-effects ordinal regression modeling for ECC performance measures. No significant differences over time (*P*>.05) were identified for the overall ECC performance score, compressions fully released, compressions with adequate depth, or compressions with the correct hand position. However, paramedics had significantly lower odds (OR 0.30, 95% CI 0.12-0.78) of achieving compressions with adequate rate at time 3 compared to time 2 (*P*=.01). Compressions were of a slower rate at time 3 (mean 85.7, SD 26.2) compared to time 2 (mean 87.8, SD 23.2) with an average difference of 2.1 fewer compressions every minute.

## Discussion

### Principal Findings

This pre- and postintervention study investigated the effectiveness of SCOPE clinical simulation resources on paramedics’ ECC. Overall, paramedic participants had high resuscitation skills before the SCOPE intervention, which were not significantly improved by training and did not degrade over time following the deployment of the manikins. However, there was one exception to this finding, namely that paramedic participants had significantly lower odds of achieving compressions with adequate rate approximately 6 to 11 months after the SCOPE intervention compared with immediately following training. Compressions were of a slower rate (average difference of 2 fewer compressions per minute) 6 to 11 months after completing training using the SCOPE resources. Despite this difference in compression rate, this did not cause significant detriment to overall ECC performance.

As no previous studies have specifically aimed to explore the impact of advanced technology manikins on the skills of registered paramedics in the prehospital setting [11,12], this research provides many important methodological lessons. In the literature with nonparamedic samples, study results suggest that simulation training may add value to learner satisfaction and process skills (proficiency, economy of movements, and minor errors) beyond that of what is provided by nonsimulation training such as face-to-face sessions and educational videos [11]. However, in this sample of high-performing paramedics, we did not find any significant benefits of clinical simulation resources for skills development and maintenance [11].

The lack of significant improvement in paramedics’ ECC skills despite the use of simulation resources could be attributed to several factors. These include the participants’ already proficient baseline skill levels, the nature of the training provided, and the complexity of ECC skills. While surveys investigating paramedics’ inclinations toward self-directed learning reveal that a significant majority would be inclined to engage in such learning if equipped with training facilities at their stations (91%) [22], we did not investigate the frequency of SCOPE use and are unable to assess its influence on the ECC performance of paramedics. Furthermore, data concerning OHCAs attended by individual paramedics in the past are unavailable from this state ambulance service. Consequently, the influence of on-the-job experience in performing cardiopulmonary resuscitation cannot be assessed and explored. However, previous research exploring methods to optimize simulation training has shown that courses using booster practice, team or group dynamics, distraction, and integrated feedback yielded significantly better skills outcomes compared to courses without these features [11].

### Limitations and Lessons Learnt

Study findings should be considered with regard to several limitations. First, paramedics demonstrated a consistently high performance both before and after the intervention, which is likely indicative of a highly skilled and capable paramedic workforce. However, it is also possible that those selected to become champions and participate in training represented a more capable subgroup of paramedics. Indeed, champions self-nominated or were selected by their colleagues and management staff because of their interest in education and training and proactive professional development behaviors. Anecdotal evidence from the services’ educational unit suggests that there may be a greater range in ECC performance in the general paramedic population from both regional and urban areas. Therefore, study findings may not be representative of the New South Wales paramedic workforce or the wider Australian paramedic workforce.

Second, the use of a pre- and poststudy design without a control group was pragmatically selected to align with the planned roll-out of the intervention across New South Wales. However, this nonexperimental observational design is not considered rigorous by the Cochrane Effective Practice and Organisation of Care group due to the risk of confounders obscuring the effect of the intervention [23]. The limitations to this study design arise from historical threat, whereby external events, unrelated to the intervention, could occur in the time between the pre- and postmeasurement, influencing resuscitation outcomes. For example, paramedic fatigue as a result of the COVID-19 pandemic may have reduced resuscitation performance at time 3, or paramedics may have been exposed to fewer OHCA over the recruitment period than typical, impacting skills maintenance. While it is possible that these study results may be an underestimate of the true positive impact of the intervention, we are unable to conclude to what extent paramedic resuscitation performance at time 3 was impacted by the intervention. Implementation of a stepped wedge design where the order of implementation of the intervention was randomized by location could overcome these limitations [24]. However, arranging SCOPE forums to fit with randomization schedules was deemed too logistically challenging, and the roll-out of the SCOPE intervention was prioritized over evaluation rigor.

Third, the study was impacted by small sample size and attrition. However, while the sample size at time 3 was low, there were no statistically significant differences in age and sex between responders and nonresponders. It is likely that a substantial proportion of participant attrition at time 3 was attributable to the high workload as a result of the widespread community transmission of COVID-19 during late 2021 and the first 3 months of 2022 when data collection was conducted. Reduced downtime during shifts and longer work hours limited paramedics’ ability to continue participating in the research study at time 3. Other factors responsible for attrition at time 3 included paramedics being relocated to metro locations without access to manikins (5%) and those unavailable due to extended leave (10%).

Fourth, no formal adjustment for multiple comparisons was performed, as the analysis of secondary outcomes was exploratory. Finally, we assessed the ECC performance of paramedics using an advanced technology simulator manikin as an indicator of paramedic capability and high-quality care. It is important to note that this one measure does not fully capture the range of skills required for high-quality Advanced Life Support care. Paramedic practice involves a multifaceted set of skills and competencies beyond ECC proficiency, including critical thinking, rapid decision-making under pressure, effective communication within the team, and the ability to manage complex clinical situations in dynamic environments [25]. Therefore, while ECC performance assessed with advanced technology simulator manikins provides valuable insight into paramedic capabilities, it is only one component of the broader spectrum of skills necessary for delivering high-quality Advanced Life Support care in diverse and challenging prehospital settings.

### Implications

Future evaluations of clinical simulation should seek to overcome the limitations of this study by using a rigorous experimental research design [23], implementing strategies to reduce attrition over time such as creating time for participation (ie, quarantined off-road time to participate in the evaluation), or rewarding participation (ie, monetary or nonmonetary initiatives including extra credit toward professional development activities) [26] and ensure a representative sample of paramedics. Given the manikins have been implemented across locations, future research could seek to explore best-practice methods to maximize the impact of the advanced technology clinical simulation manikins, as previous research in nonparamedic populations suggests incorporating booster practice, team or group dynamics, and distraction (eg, placing the manikins in real-life situations) [11]. Ideally, the OHCA case volume of participating paramedics should be collected to understand the added value of advanced technology manikins—for ambulance services where these data are not routinely collected by the service, the validity of paramedic self-report could be explored for use as a proxy measure. As has been done with other health professionals [11], future research should seek to explore and evaluate the wider potential of advanced technology manikins beyond cardiopulmonary resuscitation skills for OHCA to other scenarios such as advanced cardiac life support for adults and children, trauma life support, complex medical presentations, rapid response and other urgent clinical cases, mass casualty or terrorism response, and nonclinical skills such as working in teams, crew resource management, and graded assertiveness. In their current form, the manikins are an overt tool for assessment, and it remains unknown how ECC performance on the manikin translates to in-the-field behavior and patient outcomes. A few studies with samples of health professionals have identified positive patient effects favoring enhanced technology simulation [11,27-29]. Future research should seek to clarify the relationship between paramedics’ use of enhanced technology simulations and patient outcomes using suitable survival and mortality measures (such as return of spontaneous circulation, survival to hospital discharge rates, and postevent survival to death) [27-29].

### Conclusions

ECC performance did not significantly improve immediately after training nor did it degrade over time following the deployment of the manikins. The high baseline performance (ceiling effect) of paramedics in this sample may have impacted our ability to detect an increase in skills and performance.

## Abbreviations

ECC: external cardiac compression
OHCA: out-of-hospital cardiac arrest
OR: odds ratio
SCOPE: Simulated Curriculum for Out of Hospital Paramedic Education
